# Rituximab for the treatment of relapsing-remitting multiple sclerosis in Thailand: an economic evaluation and budget impact analysis

**DOI:** 10.1186/s12913-023-10099-1

**Published:** 2023-10-13

**Authors:** Saharat Aungsumart, Saowalak Turongkaravee, Sitaporn Youngkong, Metha Apiwattanakul, Ammarin Thakkinstian, Usa Chaikledkaew

**Affiliations:** 1 Mahidol University Health Technology Assessment (MUHTA) Graduate Program, Mahidol University, Bangkok, Thailand; 2 Neuroimmunology Unit, Department of Neurology, Neurological Institute of Thailand, Bangkok, Thailand; 3 Social and Administrative Pharmacy Division, Department of Pharmacy, Faculty of Pharmacy, Mahidol University, Bangkok, Thailand; 4 Department of Clinical Epidemiology and Biostatistics, Faculty of Medicine Ramathibodi Hospital, Mahidol University, Bangkok, Thailand

**Keywords:** Rituximab, Multiple sclerosis, Cost-utility analysis

## Abstract

**Background:**

Multiple sclerosis is an inflammatory demyelination process in the central nervous system (CNS) causing neurological disability and poor quality of life. Currently, Thai Food and Drug Administration (FDA)-approved disease-modifying therapy is costly, and most patients with multiple sclerosis are ineligible for treatment in Thailand as previous studies have challenged its cost-effectiveness. Off-label use of rituximab is inexpensive and highly effective in treating multiple sclerosis, but evidence of its cost-effectiveness in Thailand is yet to be collected.

**Methods:**

This study aimed to evaluate the cost-utility and budget impact of rituximab for multiple sclerosis treatment compared with best supportive care, the standard practice in Thailand to treat the disease. A Markov model with a one-month cycle length and lifetime horizon was applied to compare the costs and outcomes of rituximab and best supportive care based on a societal perspective. Accordingly, incremental cost-effectiveness ratios were estimated. Probabilistic and one-way sensitivity analyses were conducted to investigate parameter uncertainty. In addition, the Markov model was used to assess the 5-year budget impact from the government perspective.

**Results:**

A rituximab biosimilar demonstrated higher effectiveness and lower associated costs, compared to best supportive care, with the highest probability of being cost-effective (96%). The probability of relapse was the most sensitive parameter according to the one-way sensitivity analysis. The calculated budget impact of treating patients with multiple sclerosis in Thailand was 26,360,000 Thai baht (THB) or 844,255 United States dollars (USD) in the first fiscal year, and approximately 20,810,000–23,080,000 THB (666,608–739,388 USD) in the next four fiscal years.

**Conclusion:**

In Thailand, a rituximab biosimilar would reduce the overall costs of multiple sclerosis treatment and should, therefore, be included in the National List of Essential Medicines.

## Background

Multiple sclerosis (MS) is a chronic immune-mediated disease of the central nervous system that causes neurological disabilities, especially in young adults [1]. The incidence of MS is increasing worldwide, with two million cases reported in 2016 [2]. The prevalence and incidence of MS varies according to country and geographical areas [3]. The prevalence of MS is higher in Europe and the United States compared to that in Asia [4]. The estimated prevalence and incidence of MS in Thailand were 0.201 per 100,000 people and 0.073 per 100,000 person-years, respectively [5]. Chronic disease progression results in a significant increase in disability, leading to economic and social burdens [6]. Therefore, most patients who are unable to access MS treatment are affected by neurological disabilities, poor quality of life, and an additional socioeconomic burden [7].

Disease-modifying therapy (DMT), which includes injectable platform therapies, oral medication, or monoclonal antibodies, has been proven to prevent relapse and patient disabilities [8]. However, the cost associated with DMT is the most critical barrier preventing access to treatment [9]. The price of DMT approved by the Thai Food and Drug Administration (FDA) ranges from 39,544 to 61,714 Thai baht (THB) per month, which is equivalent to 1,041 − 1,625 United States dollars (USD), whereas the average Gross National Income (GNI) per capita of the Thai population is only 18,888 THB per month (497 USD). According to a recent survey by the Multiple Sclerosis International Federation (MSIF) [9], out of all the 102 countries where rituximab has been approved for the treatment of lymphoma and rheumatoid arthritis, 70 of them (including Thailand) have adopted it as off-label treatment for MS, because the benefits were considered to outweigh the need for regulatory approval [10]. Previous studies from several countries have suggested that rituximab is not only safe and highly effective in the control of MS progression [11–20], but it also has a lower cost than the currently approved DMT. The approximate annual cost of MS therapy in Thailand with the original rituximab is 100,000 THB (2,633 USD). However, a rituximab biosimilar is readily available in the country, and its efficacy has been shown to be similar to the original drug in the treatment of diffuse large B-cell lymphoma and severe rheumatoid arthritis [21, 22]. The current cost of therapy for the same period of time with the rituximab biosimilar is only 27,000 THB (711 USD).

Until recently, only a small minority of patients with MS in Thailand could get access to FDA-approved DMTs or to rituximab therapy, as neither of these treatments have been included in the National List of Essential Medicines (NLEM). This pharmaceutical reimbursement list is used by all Thai health insurance schemes, including the Civil Servant Medical Benefit Scheme (CSMBS) for government officers and their dependents (9%), the Social Security Scheme (SSS) for private employees (16%), and the Universal Coverage Scheme (UCS) for the general population (75%) [23]. The decision-making process to add a drug to the NLEM, especially one associated with high costs, requires the previous collection of efficacy, safety, cost-effectiveness, and budget impact information [24]. Although three cost-utility analyses of DMT in Thai patients with MS suggested that DMT would not be cost-effective at the willingness-to-pay (WTP) threshold estimated for the country of 160,000 THB (4,214 USD) per quality adjusted life year (QALY) gained [25–27], the cost-effectiveness of rituximab as an alternative has not yet been investigated. Therefore, the objective of this study was to evaluate the cost-utility of rituximab compared with best supportive care (BSC), the current standard practice for the treatment of patients with MS in Thailand. In addition, the budget impact for patients with MS if rituximab were to be included in the NLEM was estimated. The results of this study could inform decision-making on the potential inclusion of rituximab in the NLEM.

## Methods

### Study design

A Markov model was developed to evaluate the cost-utility of rituximab in the treatment of MS compared with BSC based on a societal perspective, as recommended by the guidelines for Health Technology Assessment (HTA) in Thailand [28]. Target population was the cohort of newly diagnosed relapsing-remitting multiple sclerosis (RRMS) patients above 18 years of age.

### Interventions and comparator

Both rituximab and a rituximab biosimilar were included in the analysis as alternative treatment options. The dose of rituximab and the biosimilar drug was based on a standard fixed-dose regimen starting with an induction injection of 1000 mg at day 1 and day 14, repeated every six months until patients reached the Expanded Disability Status Scale (EDSS) score of 6.0. The difference between rituximab and biosimilar drugs in our study was the annual cost of the medication i.e., 100,000 THB (2,633 USD) versus 27,000 THB (711 USD), respectively.

### Decision-analytic model

#### Model structure

**Fig. 1:**
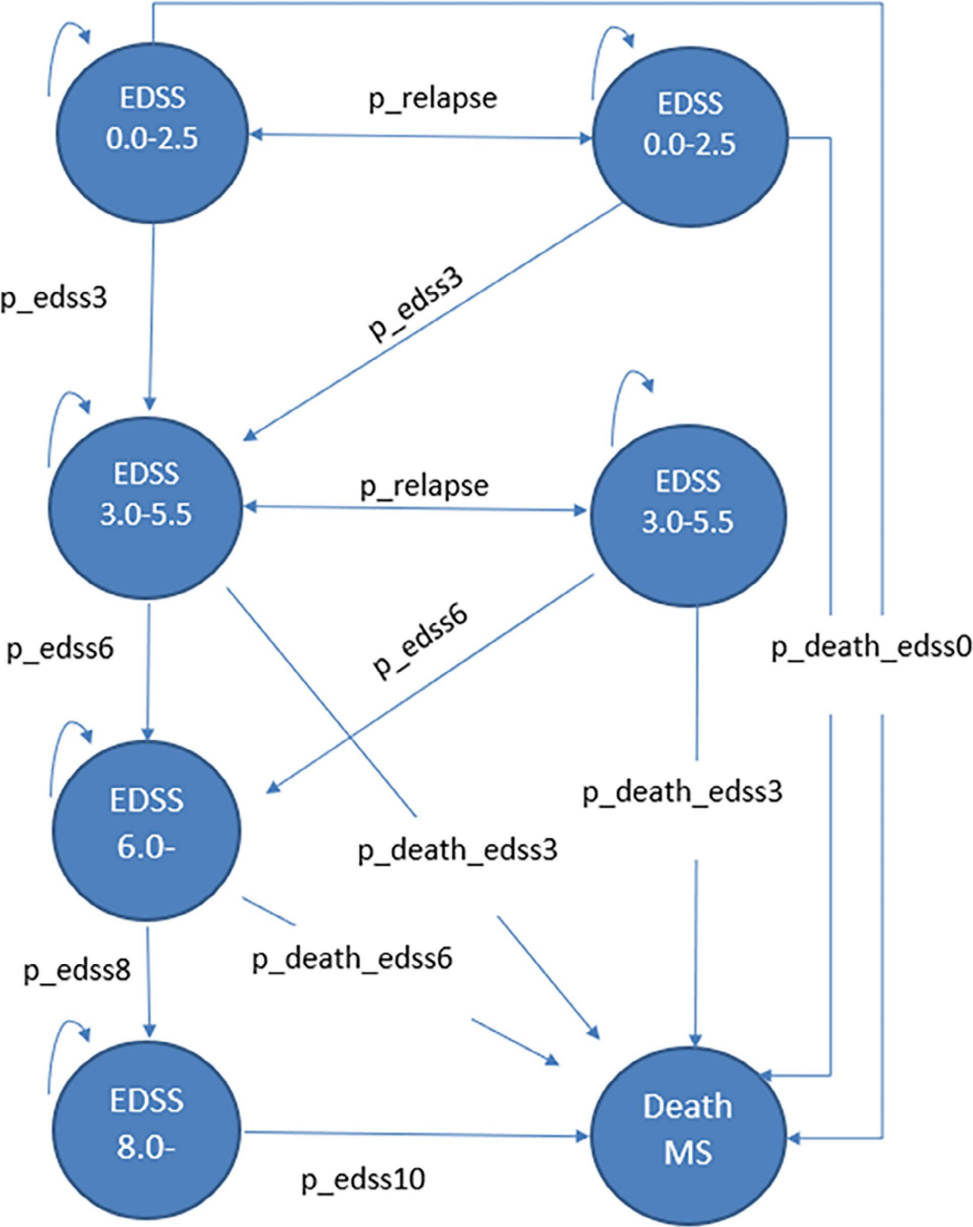
Schematic Markov model for multiple sclerosis (MS) treatments. MS = multiple sclerosis, EDSS = Expanded Disability Status Scale, p_relapse = transition probability of progressing from EDSS 0.0–2.5 to relapse EDSS 0.0–2.5 (and vice-versa), or; transition probability of progressing from EDSS 3.0–5.5 to relapse EDSS 3.0–5.5 (and vice-versa); p_edss3, p_edss6, and p_edss 8 = transition probabilities of progressing from earlier EDSS health state to EDSS 3.0–5.5, to EDSS 6.0–7.5, and EDSS 8.0–9.5, respectively; p_death_edss0, p_death_edss3, or p_death_edss6; and p_edss10 = transition probabilities of progressing to death from EDSS or Relapse EDSS 0.0–2.5, from EDSS or Relapse EDSS 3.0–5.5, from EDSS 6.0–7.5, and from EDSS 8.0–9.5, respectively

The Markov model used in this study was modified from a previously published cost-utility analysis of MS treatment in Thailand [26]. Face validity of the model was assessed through consultations with two clinicians with expertise in MS disease progression and two health economists with cost-effectiveness modeling expertise. These experts provided judgments on the appropriateness of the Markov model and input parameters, model structural uncertainty, computerized model, data sources, and model outcomes. Figure 1 shows the model structure that simulated MS patients’ clinical courses, and estimated costs and health outcomes over a lifetime horizon with a cycle length of one month. Five primary health states based on the EDSS were considered, including (i) patients without disability or mild disability (EDSS 0.0-2.5), (ii) patients with moderate disability or ambulatory limitation (EDSS 3.0-5.5), (iii) patients with severe disability who required walking aids or wheelchairs (EDSS 6.0-7.5), (iv) patients confined to bed (EDSS 8.0–9.5) and (v) deceased MS patients. Two additional health states related to relapse were defined, including (vi) patients without disability or mild disability (EDSS 0.0–2.5) and (vii) patients with moderate disability or ambulatory limitation (EDSS 3.0-5.5). The arrows in Fig. 1 denote permissible transitions between health states within the model.

#### Model assumptions

There are several assumptions in this model. First, it is assumed that all patients would experience all the EDSS health states. Second, patients would terminate treatment after reaching the EDSS 6.0-7.5 health state, which indicates progressive disease. According to current clinical practice guidelines in Thailand, patients who progress to a health state with an EDSS above 6.0 would receive BSC as standard treatment. Third, the same transition probability of relapse was applied to patients with EDSS 0.0-2.5 and EDSS 3.0-5.5. Fourth, the probability of relapse was considered diminished after patients with EDSS 6.0-7.5 and EDSS 8.0-9.5. Fifth, either rituximab or a biosimilar drug was considered to be first-line treatments, and both were assumed to have similar efficacy. However, the use of rituximab as a first-line agent may not be standard clinical practice in some settings as immunosuppressive agents such as azathioprine or methotrexate, which have lower associated costs, may be preferred in patients with MS [9]. Nevertheless, previous studies have shown that the clinical efficacy of immunosuppressive therapy for MS is lower compared to that of rituximab [29, 30]. Therefore, in this study, we excluded immunosuppressive agents. Sixth, patients could not transition to a health state associated with a lower EDSS, reflecting the chronic progression of MS over time. Seventh, the model did not specify the clinical point at which patients progressed to secondary progressive MS.

#### Model parameters

The input parameters used in the model were classified into four groups, as follows: (1) epidemiological data and transition probabilities, (2) effectiveness of treatment, (3) cost data, and (4) utility data. The parameter values are presented in Table 1.

#### Epidemiological data and transition probabilities

Based on our systematic review, the transition probabilities for MS progression and relapse were obtained from published studies conducted on a prospective longitudinal cohort of MS patients with long follow up period [31–33]. The transition probability for MS relapse was retrieved from a previous study in which MS patients were prospectively followed up for one to five years [31], and those of disease progression for MS patients receiving BSC were obtained from a cohort study that prospectively investigated time to progression classified by EDSS scores among 1,099 MS patients receiving care at University Hospital, London, Canada, between 1972 and 1984 [32]. In addition, the transition probabilities of death due to MS from EDSS 0.0–2.5 and EDSS 3.0–5.5 were obtained from a study in which 806 RRMS patients receiving treatment at the London Multiple Sclerosis Clinic in Canada were followed up for 28 years [33]. Data on the efficacy of rituximab to prevent relapse compared with placebo in RRMS were obtained from a single phase II randomized controlled trial in 2008 [34]. In addition, the probability of disability progression in rituximab-treated MS patients was retrieved from a phase III randomized controlled trial that represented the most recent study comparing rituximab with dimethyl fumarate [35]. The efficacy of rituximab to prevent relapse was not derived from this study because our specific objective was to compare the effect of rituximab therapy with that of no DMT. Finally, we assumed that the efficacy and safety of rituximab biosimilar were similar to those reported in the original clinical trials for rituximab, since no data involving the direct comparison of these parameters between rituximab and biosimilar drugs in the treatment of RRMS was available.

#### Cost data

Based on a societal perspective, both direct medical and non-medical costs corresponding to patients with MS in Thailand were included and obtained from a previously published cross-sectional study of patients with MS recruited between March 1, 2011 and September 30, 2014 [26]. Direct non-medical costs incurred by MS patients and their families included costs of transportation, food, accommodation, facility modification, required equipment, as well as formal and informal care [26]. Direct medical costs included all treatment costs except those of DMT. The cost of rituximab was obtained from the reference price database of the Division of National Drug Information (NDI), Ministry of Public Health [36], whereas the cost of biosimilar rituximab was retrieved from the hospital drug list of the Neurological Institute of Thailand [37]. The administrative cost of rituximab therapy was based on a standard fixed-dose regimen starting with induction rituximab 1000 mg at day1 and day 14, repeated every six months until patients reached an EDSS score of 6.0. Therefore, the cost of rituximab in the model was the average cost during a five-year administration period. Data on rituximab-related complications in patients with MS, including infusion-related reactions, severe and non-severe infections, and agranulocytosis, were obtained from a previously published meta-analysis [11]. In addition, the costs of each rituximab-related complication and rescue therapy were obtained from the Standard Cost List for Health Technology Assessment, which has been developed since 2014. The list contains a reference unit cost including direct medical, direct non-medical, and indirect costs and has been previously applied in cost analysis to better reflect costs in Thailand [38]. The consumer price index was applied to adjust all the costs to 2022 values, and the exchange rate of 37.97 THB to one USD was used.

#### Utility data

Following a systematic search, we obtained the utility data from a previous study by Siritho et al. [7], as this was the only cross-sectional study reporting the utility for Thai patients with MS classified by EDSS scores that we were able to identify. However, due to the fact that this study excluded Thai MS patients with relapse [7], we adopted the disutility due to MS relapse from a previously published study in the UK [39]. Health outcomes are presented in terms of QALYs, multiplication of life years (LYs), and utility scores.

#### Discount rate

Future costs and outcomes were adjusted to present values using a discount rate of 3%, as recommended by the guidelines for HTA in Thailand [28].

#### Results presentation

Total cost, LYs and QALYs associated to the three alternatives (BSC, rituximab and biosimilar drug) were estimated. The incremental cost-effectiveness ratio (ICER) was calculated by dividing the incremental cost by the incremental QALYs associated with rituximab or the biosimilar drug in reference to BSC. Accordingly, the ICER was presented as cost (in THB) per QALY gained. The ICER results were compared with a WTP threshold of 160,000 THB (5,128 USD) per QALY gained, as recommended by the Subcommittee for the Development of the NLEM [28]. Data were analyzed using Microsoft Excel (Microsoft Corporation, Redmond, WA, USA).

**Table 1:** Input parameters used in the model

Cost and input parameter	Distribution	Mean	Standard deviation	Reference
Discounting				
Discount rate for costs (%)		3	(0–6)	
Discount rate for outcomes (%)		3	(0–6)	
Transition probability of BSC				
Progression from EDSS 0.0–2.5 to EDSS 3.0–5.5	Beta	0.0075	0.0046	[31]
Progression from EDSS 3.0–5.5 to EDSS 6.0–7.5	Beta	0.0079	0.0021	[31]
Progression from EDSS 6.0–7.5 to EDSS 8.0–9.5	Beta	0.0018	0.0018	[32]
Progression from EDSS 8.0–9.5 to death	Beta	0.0017	0.0017	[31]
Relapse	Beta	0.0755	0.0755	[32]
Death due to MS from EDSS 0.0–2.5	Beta	0.0009	0.0009	[33]
Death due to MS from EDSS 3.0–5.5	Beta	0.0011	0.0011	[33]
Transitional probability for rituximab				
Relapse	Beta	0.4220	0.1727	[34]
Progress EDSS 0.0–2.5 to EDSS 3.0–5.5	Beta	0.0041	0.0008	[35]
Progress EDSS 3.0–5.5 to EDSS 6.0–7.5	Beta	0.0041	0.0008	[35]
Adverse drug reaction				
Infusion-related reaction	Beta	0.1880	0.0376	[11]
Minor infection	Beta	0.1690	0.0338	[11]
Agranulocytosis	Beta	0.0111	0.0022	[11]
Hospitalization pneumonia	Beta	0.0065	0.0013	[11]
Cost variable (THB)				
OPD direct medical cost for EDSS 0.0–2.5	gamma	1317.70	131.84	[26]
OPD direct medical cost for EDSS 3.0-5.5	gamma	2408.77	349.20	[26]
OPD direct medical cost for EDSS 6.0-7.5	gamma	2202.10	256.56	[26]
OPD direct medical cost for EDSS 8.0-9.5	gamma	2013.25	260.12	[26]
Direct medical cost for EDSS 0.0–2.5 with relapse	gamma	40585.63	4407.76	[26]
Direct medical cost for EDSS 3.0-5.5 with relapse	gamma	71144.23	7710.91	[26]
Direct medical cost for EDSS 6.0-7.5 with relapse	gamma	80814.94	9688.53	[26]
Direct medical cost for EDSS 8.0-9.5 with relapse	gamma	55626.19	7917.58	[26]
Direct non-medical costs for EDSS 0.0–2.5	gamma	2686.70	734.03	[26]
Direct non-medical costs for EDSS 3.0-5.5	gamma	2975.33	1172.32	[26]
Direct non-medical costs for EDSS 6.0-7.5	gamma	10846.59	1949.11	[26]
Direct non-medical costs for EDSS 8.0-9.5	gamma	15061.94	2782.91	[26]
Cost of rituximab average per month				
Rituximab (24,182 THB/500 mg vial)	gamma	8,236	1,647	[36]
Rituximab biosimilar (8,667 THB/500 mg vial)	gamma	2,967	590	[37]
Cost of rituximab administration per month	gamma	506.34	101	[38]
Treatment cost of adverse drug reaction				
Infusion-related reaction	gamma	1465.12	293	[38]
Minor infection	gamma	4999.92	1,000	[38]
Agranulocytosis	gamma	23952.00	4,790	[38]
Hospitalization due to pneumonia	gamma	9118.03	1,824	[38]
Utility of patients with MS				
EDSS 0.0-2.5	Beta	0.600	0.02	[7]
EDSS 0.0-2.5 with relapse	Beta	0.524	0.10	[7, 39]
EDSS 3.0-5.5	Beta	0.490	0.04	[7]
EDSS 3.0_5.5 with relapse	Beta	0.414	0.08	[7, 39]
EDSS 6.0-7.5	Beta	0.170	0.06	[7]
EDSS 8.0-9.5	Beta	0.026	0.01	[7]

### Uncertainty analysis

#### Parameter uncertainty

One-way and probabilistic sensitivity analyses (PSA) were performed to address the sensitivity of the model to each of the input parameters. One-way sensitivity analysis was performed by varying each input parameter within its 95% CI, and a Tornado diagram was used to present the resulting range of ICER values. In addition, PSA was performed to simultaneously evaluate the uncertainty of all parameters using a 1,000 Monte Carlo simulation. Consequently, the cost-effectiveness acceptability curve (CEAC) was used to determine the probabilities of each alternative being cost-effective relative to a specific WTP threshold.

#### Budget impact analysis

A budget impact analysis from the government’s perspective was also conducted to assess the affordability of offering rituximab therapy to patients with MS. A Markov model-based approach was used to calculate the budget impact of replacing BSC with rituximab therapy in patients with MS from a governmental perspective during five consecutive fiscal years. The total budget was calculated by multiplying the number of patients by the direct medical costs per patient. We estimated the number of patients with MS in 2021 using the data on prevalence (0.201 per 100,000 individuals) and incidence (0.073 per 100,000 individuals) of MS in Thailand reported in 2012 [5], yielding approximately 572 patients with MS in 2021 and 51 new cases per year. The budget impact was estimated under the assumptions that no discounts were applied, the incidence rate of MS was stable, and the treatment coverage rate was 90%.

## Results

### Cost-utility analysis

Markov model simulated a scenario in which MS patients received BSC, rituximab, or a biosimilar drug for a period of 30 years. The estimated total costs, LYs, QALYs, and ICER are shown in Table 2. Rituximab and its biosimilar increased both LYs and QALYs gained by 0.34 and 1.79, respectively. However, rituximab increased the cost of treatment by 779,984 THB (20,542 USD), whereas in the case of the biosimilar drug the cost decreased by 237,469 THB (6,254 USD) compared to that of BSC. The ICER of rituximab was approximately 434,666 THB (11,447 USD) per QALY gained, and 2,265,882 THB (59,676 USD) per LY gained. On the other hand, the ICER of the rituximab biosimilar showed a dominant result indicating more effectiveness and a lower cost compared with BSC.

**Table 2:** Cost-effectiveness results

Medication	Total cost THB (USD)	Total effectiveness		Incremental cost THB (USD)	Incremental effectiveness		ICER THB (USD)	
		LYs	QALYs		LYs	QALYs	LYs	QALYs
BSC	3,099,205 (81,622)	20.33	7.86					
Rituximab	3,879,189 (102,165)	20.67	9.65	779,984 (20,542)	0.34	1.79	2,265,882 (59,676)	434,666 (11,447)
Rituximab biosimilar	2,861,736 (75,368)	20.67	9.65	-237,469 (-6,254)	0.34	1.79	Dominant*	Dominant*

*Dominant indicates a lower cost but higher effectiveness

### Uncertainty analysis

**Fig. 2:**
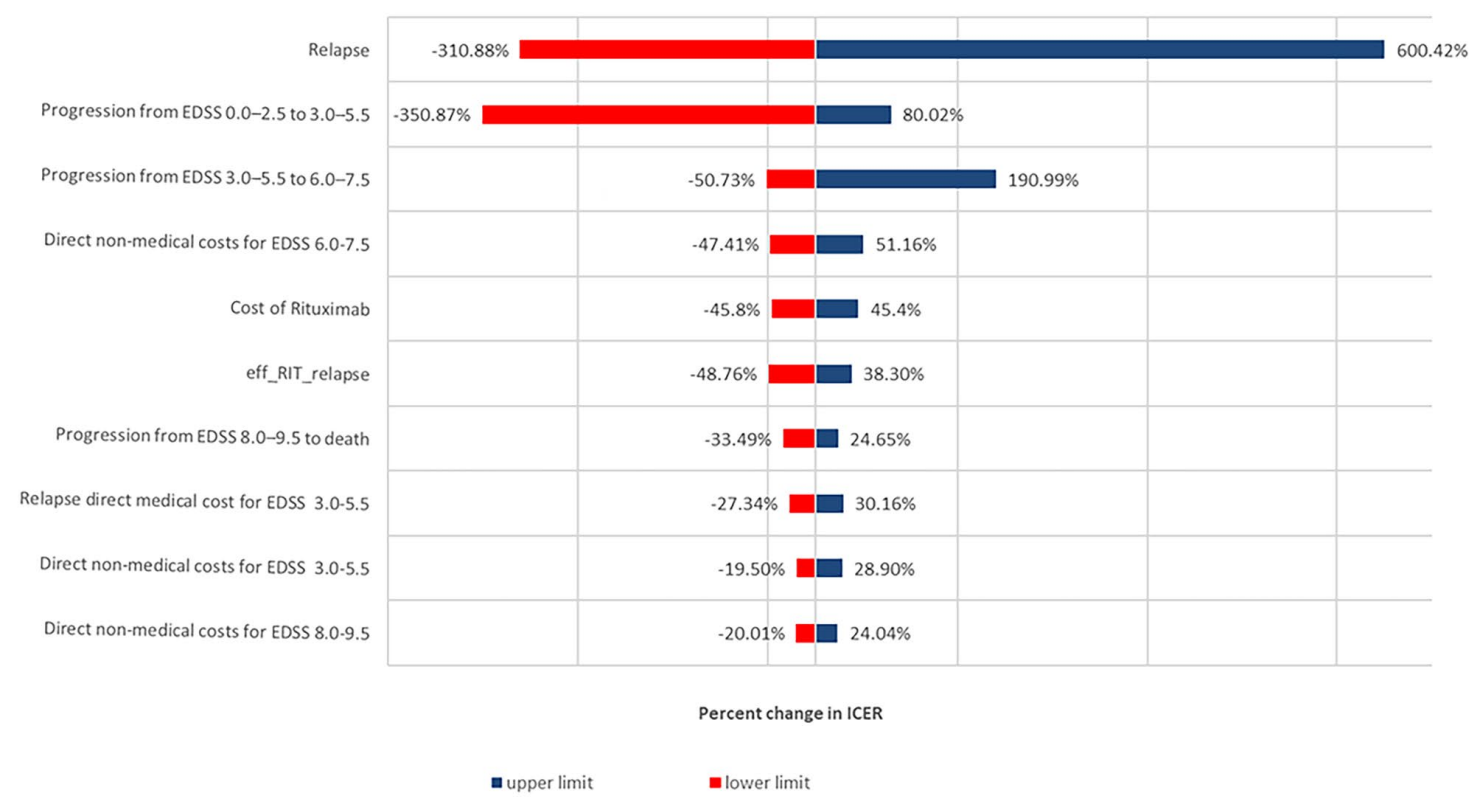
One-way sensitivity analysis results for rituximab biosimilar

The one-way sensitivity analysis results for the rituximab biosimilar are presented in Fig. 2. The ICER was most sensitive to the probability of relapse, followed by the probability of progression from EDSS 0.0-2.5 to EDSS 3.0-5.5, direct non-medical costs for EDSS 6.0-7.5, the probability of progression from EDSS 3.0–5.5 to EDSS 6.0–7.5, direct non-medical cost for EDSS 6.0-7.5, the cost of rituximab therapy (a 20% change from the base case), and the efficacy of rituximab to prevent relapse.

**Fig. 3:**
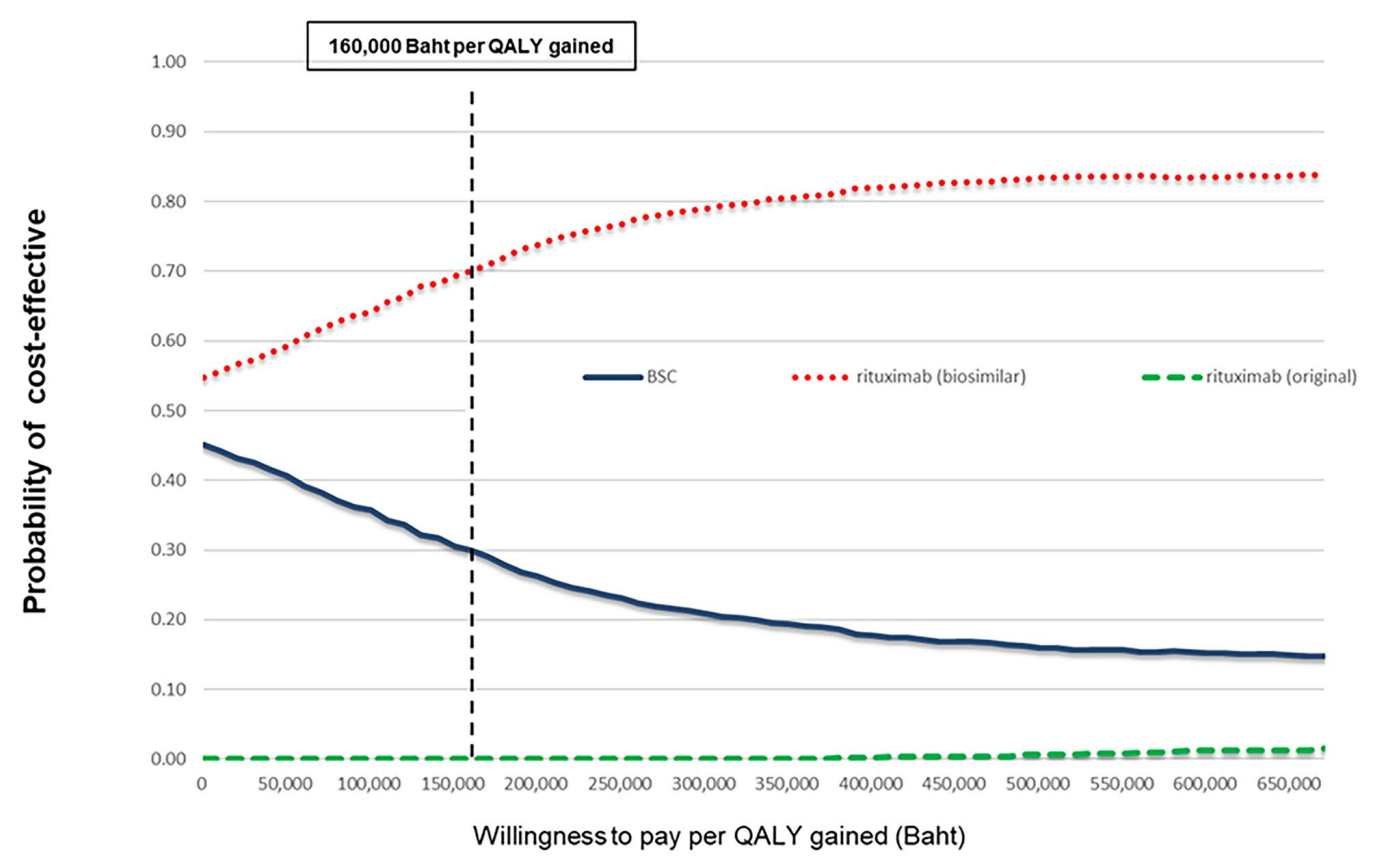
Cost-effectiveness acceptability curves. BSC: best supportive care

Figure 3 shows the cost-effectiveness acceptability curves for all treatment options. The rituximab biosimilar had the highest probability of being cost-effective (70%) at a willingness-to-pay (WTP) threshold of 160,000 THB (4,214 USD), whereas that of BSC was approximately 30%.

### Budget impact analysis

**Fig. 4:**
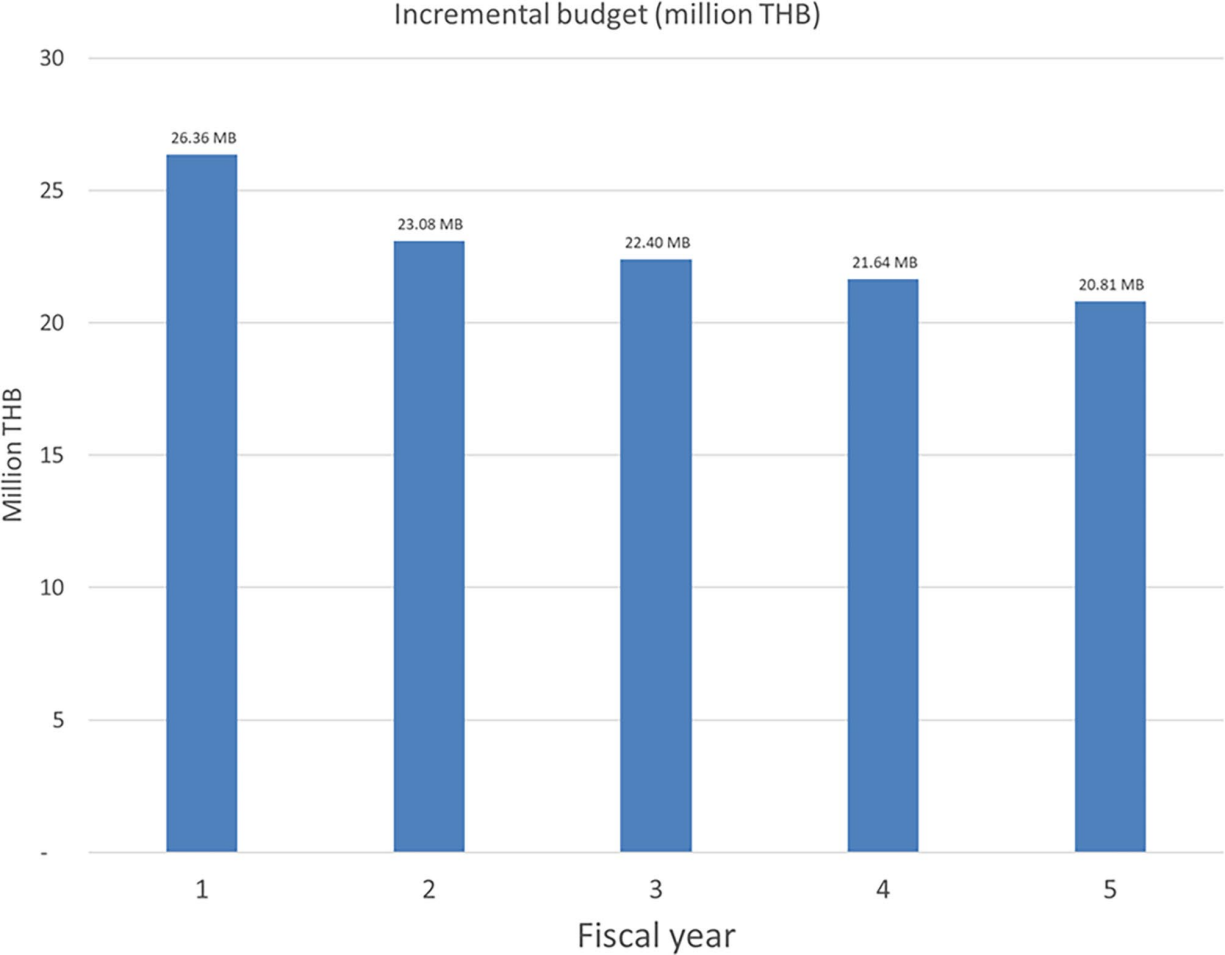
Estimated budget impact during five consecutive fiscal years

The budget impact from a governmental perspective over five years is shown in Fig. 4. The incremental budget was approximately 26,360,000 THB (844,255 USD), followed by 23,080,000 THB (739,388 USD), 22,400,000 THB (717,603 USD), 21,640,000 THB (693,223 USD), and 20,810,000 THB (666,608 USD) during five consecutive fiscal years, respectively.

## Discussion

Regardless of the evidence indicating that DMT can prevent hospitalization and relapse as well as decrease disability in MS patients [8], most MS patients in Thailand are still unable to afford these expensive treatments, especially in low- or low-to- middle-income countries. In Thailand, DMT has not been included in the NLEM, as previous cost-effectiveness studies have suggested that it is not cost-effective. Currently, rituximab in both original and biosimilar forms has been approved for the treatment of lymphoma and rheumatoid arthritis but not MS in Thailand. Nevertheless, it has been used as an off-label treatment for patients with MS in clinical practice. Given that no cost-effectiveness information regarding the use of rituximab for MS treatment specific to Thailand has been reported, the drug has not been included in the NLEM, a pharmaceutical reimbursement list for all health insurance schemes in the country. Therefore, the Subcommittee for the Development of NLEM requested cost-effectiveness information on rituximab in order to consider whether it should be included into the NLEM. To the best of our knowledge, this study is the first to evaluate the cost-utility of rituximab compared to BSC in Thai patients with MS.

Our study suggests that the rituximab biosimilar would be dominant or cost-saving, indicating a lower cost but a higher effectiveness compared to BSC for MS treatment in Thailand. This study also demonstrated that the rituximab biosimilar has a high probability of being cost-effective compared with BSC, the current standard of treatment in the Thai healthcare system. The estimated budget impact of the rituximab biosimilar in patients with MS was approximately 25,000,000 THB per year during five consecutive fiscal years. In contrast, the original rituximab would not be cost-effective in the Thai context compared to BSC. Consequently, the results of this study could help inform policymakers on the potential advantages of including the rituximab biosimilar in the NLEM for MS treatment.

In support of this recommendation, it is worth mentioning that the use of rituximab in patients with MS is a common clinical practice in developed countries. For example, the efficacy of rituximab in reducing MS relapse has been supported by phase I and II clinical trials since 2008 [34, 40]. However, although phase III clinical trials on the effectiveness of this drug to manage patients with MS have not been performed, it has nevertheless been regularly prescribed to these patients during the past five years in several countries, including Sweden [18], the US [16, 20], Spain [17], Switzerland [15], France [12], Italy [13], Germany [41] and Lebanon [14]. Notably, based on the clinical guidelines of the Middle East and North Africa Committee (MENACTRIMS) Consensus, rituximab is recommended as an off-label treatment for patients with highly active MS disease. Moreover, it should be administered as an escalation treatment to patients with all levels of MS in special populations such as refugees or in countries where other proper MS treatments are not accessible [42]. In addition, according to the most recently updated guidelines from the American Academy of Neurology (AAN) published in 2018, rituximab is possibly more effective than placebo in reducing the risk of relapse at one year for patients with RRMS [43]. The efficacy of rituximab compared with the FDA-approved DMT, (i.e., dimethyl fumarate) has been confirmed through a phase III randomized controlled trial [35]. Therefore, this study can be considered as additional evidence on the advantages of including a rituximab biosimilar in the NLEM for the specific purpose of MS treatment. However, several considerations should be considered by physicians before prescribing rituximab or a biosimilar drug to MS patients, including side effects, disease activity, and off-label medication use in the treatment of MS.

Furthermore, the one-way sensitivity analysis indicated that the most sensitive parameter was the probability of relapse. The results indicate that treating patients with MS that experience frequent relapses may be a cost-saving intervention compared with BSC. In contrast, rituximab biosimilar would not be cost-effective in patients with few relapses. This could be explained by the fact that rituximab is particularly effective in patients with high disease activity. This is in line with the recommendations of several MS treatment guidelines [43, 44], as physicians tend to use DMT only in MS patients with active disease.

Some limitations of this study need to be addressed. First, the transitional probabilities of rituximab to prevent relapse and disability progression were obtained from only two randomized controlled studies, which were the best available evidences on the effect of rituximab in patients with MS to date [34, 35]. In 2008, a phase II double-blind randomized controlled trial [34] revealed the safety and efficacy of rituximab compared with placebo in patients with RRMS; more recently, a phase III randomized controlled trial from 2022 demonstrated that rituximab is safe and effective compared with dimethyl fumarate [35]. Second, we assumed that rituximab and its biosimilar had similar efficacy for MS treatment, since no head-to-head comparison between the two for the specific purpose of MS treatment is available in the literature. Nevertheless, the efficacy of the rituximab biosimilar in patients with diffuse large B-cell lymphoma [21] and severe rheumatoid arthritis [22] has been shown to be similar in pharmacokinetic studies. Third, due to the lack of data from Thailand, all transition probabilities for each health state were obtained from prospective longitudinal cohort studies published in other countries. Therefore, these parameters should be collected from local data in future studies. Fourth, as there is no data on disutility due to MS relapse available in Thailand, we obtained the relevant data from a UK study [39], which is the only available source to date. Thus, the utility of MS patients with relapse in Thailand should be the focus of future studies. Finally, it should be noted that as there is no clinical data of MS patients in Thailand, we could not validate the prediction of the model. However, we performed model validation via the face validity method through consultations with clinical experts and health economists on the appropriateness of model structure, parameters used, and data sources. Model validation should be performed in future studies. Moreover, we did not investigate how appropriate the cut-off scores were in the Markov model, which warrants further research.

In conclusion, this study demonstrated that, in the context of the Thailand healthcare system, treatment with a rituximab biosimilar was cost-saving and exhibited a high probability of being cost-effective when compared with the current practice. The estimated budget impact of treating patients with active RRMS were 26,360,000 THB (844,255 USD), followed by 23,080,000 THB (739,388 USD), 22,400,000 THB (717,603 USD), 21,640,000 THB (693,223 USD), and 20,810,000 THB (666,608 USD) during five consecutive fiscal years, respectively. This study may encourage policy decision makers to extend the indications of including rituximab biosimilar in the NLEM to treat patients with MS.

## Data Availability

The datasets used and analysed during the current study are available from the corresponding author on reasonable request.

## List of Abbreviations

BSC: Best Supportive Care
CEAC: cost-effectiveness acceptability curve
CNS: Central Nervous System
CSMBS: Civil Servant Medical Benefit Scheme
DMT: Disease-modifying therapy
EDSS: Expanded Disability Status Scale
FDA: Food and Drug Administration
HTA: Health Technology Assessment
LYs: Life years
MSIF: Multiple Sclerosis International Federation
NDI: Division of National Drug Information
NLEM: National List of Essential Medicines
QALY: Quality-adjusted life years
SSS: Social Security Scheme
THB: Thai baht
UCS: Universal Coverage Scheme
USD: United States dollars
WTP: Willingness-to-pay
